# Urine and fecal incontinence prevalence is increased in later born 81-year old cohorts in the Swedish general elder population: data from the Good Aging in Skåne study (GÅS)

**DOI:** 10.1186/s12877-026-06999-6

**Published:** 2026-02-18

**Authors:** Sölve Elmståhl, Valgerdur Thorsteinsdottir

**Affiliations:** Department of Clinical Sciences in Malmö, Division of Geriatric Medicine, Lund University, Skåne University Hospital, Jan Waldenströmsgata 35, Malmö, 205 02 Sweden

**Keywords:** Age, Epidemiology, Urine, Fecal, Incontinence

## Abstract

**Background:**

The prevalence of urinary incontinence (UI) and fecal incontinence (FI) are conditions that increase with age. Apart from few larger studies data on prevalence and severity of UI and FI is scarce, especially among the > 80 yrs. and data of birth cohort effects is lacking.

**Methods:**

The purpose of this study was to evaluate birth cohort effects on prevalence of UI and FI in the general older population. 6 714 subjects, 60 to 93 yrs were included during 2001 to 2019 from the ongoing general population study, Good Aging in Skåne (GÅS), part of the Swedish National study on Aging and Care (SNAC). The sample included four different birth cohorts aged 60 and 81 years at investigation and born 18 year apart with medical examination and questionnaire on prevalence and severity of UI and FI.

**Results:**

Prevalence of UI in men ranged from 10% aged 60–69 yrs. to 28% > 80 yrs and corresponding values for women ranged from 21% to 42%. Prevalence of FI in men ranged from 2% to 8% >80 yrs., and corresponding values for women ranged from 4% to 14% among age groups. Birth cohort effects were noted with higher prevalence of UI reported by later born (6 to 18 yrs later) men and women aged 81 yrs., OR 2.95 and 1.61, respectively, adjusted for stroke and dementia. Any UI for 81-year old men increased from 11% year 2001 to 25% among the 2019 cohort and UI the past 3 months among 81-year old women increased from 32% to 42% in the latest born cohort (*p* < 0.05). FI the past 3 months among 81-year old men increased from 4% to 11% in the latest born cohort (OR 2.95, *p* = 0.056). Only one out of three men and one out of two women with UI used aids.

**Conclusions:**

UI and FI is highly prevalent in community dwelling elder Swedish population. A birth cohort effect is noted with increasing prevalence of UI and FI among 81-year old subjects in the later born cohorts, despite a better health in these birth cohorts.

**Supplementary Information:**

The online version contains supplementary material available at 10.1186/s12877-026-06999-6.

## Introduction

Urinary incontinence (UI) and fecal incontinence (FI) are conditions that increase in prevalence with age and can have negative impact on quality of life, cause social isolation and can be impetus for long-term care placement [1, 2]. UI is defined by the International Continence Society as any involuntary leakage of urine [3]. FI is commonly defined as the involuntary loss of liquid or solid feces or mucus [4]. UI is more common in women, in frail older patients and tends to be more severe in those with more advanced frailty [3, 5].

Despite UI being a common condition, which can increase overall morbidity, mortality, and health care costs, it is underdiagnosed and undertreated and UI has been identified as a health priority by the World Health Organization [5, 6]. In a recent meta-analysis of twenty-nine studies where UI was defined as any leakage, the prevalence of UI in women older than 55 years varied from 25 to 45% [7]. Associated factors being age, obesity, diabetes, education, delivery rank, hypertension, smoking, and urinary infections [7]. A corresponding study on pooled prevalence of male UI varied from 21% to 32% aged 65 years and older where UI was defined as daily, weekly, or monthly episodes of urine leakage [8]. Less studies have reported prevalence of UI among community dwelling elder (> 80 yrs.) subjects [9, 10].

Fecal incontinence can be a challenging condition and has high psychosocial and economic impact. Most subjects do not receive care though effective treatment options is available and treating underlying bowel disturbances are one of the most effective first-line treatments [11]. The prevalence of FI varies widely in the literature, ranging from 2% to 21% and a large US National Survey from 2015 of community dwelling individuals above 65 years of age reported 18% ever experienced FI and 7% within the past 7 days [12].

Studies on health trends have become increasingly important and the importance of different aging trajectories has been recognized. A better understanding of the birth cohort effect and the ‘Flynn effect’ that later-born cohorts outperform earlier born cohorts for example in cognitive abilities is essential for improving health throughout life [13]. Older adults born in different years differ in their health later in life and this could in part be explained by difference in exposure during the life course as well as introduction of new treatment management of several medical conditions like diabetes, stroke, hypertension, cardiovascular diseases [14]. The experience of a cohort early in life can shape exposures and risk factors that are carried forward as the group ages. In the US birth cohort (1900–2000) study of more than 179 million deaths, the all-cause mortality rates declined from 1969 to 2020 [15]. Several different cohort studies have reported improved both physical and cognitive functioning in later-born cohorts of 60-, 70- and 81-year-old community dwelling subjects born 10 to 12 years apart with improvements in the later-born cohorts, reduced mortality, substantially reduction in incident diseases like CVD and stroke [13, 14, 16–21]. A systematic review of all population-based stroke incidence studies reported a 28% decrease in stroke between 1990 and 2010 [22]. The noted better health in general, and better both physical and cognitive functioning in later born cohorts support the assumption that birth cohort could be used as a surrogate for health status. This applies especially for the so-called Baby Boom cohorts born between 1940thies to 1950thies, all exposed to social, economic and health care changes at about the same point in their lives [14].

However, to our knowledge no previous study has investigated whether similar birth cohort trends with improved health also apply to lower prevalence of UI and FI in later born cohorts. Recent data on prevalence of UI and FI among elder subjects are also scarce aside from a couple of large studies on prevalence of UI in women.

The aim of this study was to determine the prevalence and severity of urinary and fecal incontinence in community dwelling elder population stratified by age and gender and secondly to describe any birth cohort effects on prevalence of UI and FI in four different birth cohorts of individuals 60 and 81 years old 18 years apart that underwent assessment in 2001, 2007, 2013 and 2019.

## Materials and methods

### Study population

The participants were recruited from an ongoing longitudinal Swedish general population study, Good Aging in Scania (GÅS) which is part of the Swedish National study on Aging and Care (SNAC) [23]. The survey included a separate enrolment of participants of the same age examined in person at a research centre during four time periods for birth cohort comparisons. The first recruiting period year 2001–2004 included both men and women in nine age cohorts: 60, 66, 72, 78, 81, 84, 87, 90 and 93 years. Of 5 370 individuals that were invited to participate 2 931 fulfilled examinations (response rate 60%).

Three additional cohorts of men and women aged 60 and 81 years at time for examination were included year 2007 (*n* = 1 523 subjects), year 2013 (1 350 subjects) and year 2019 (1 032 subjects). The participation rate for the 60-year-old age group were for the four cohorts from year 2001 to year 2019 as follows: 63%, 72%, 75% and 73%. The corresponding figures for the 81-year-old age group were 62%, 70%, 89% and 60%.

Individuals were randomly selected from the National Population Register and invited by letter to an outpatient research clinic. Home visits were offered to those who were unable to visit the study centre to avoid selection bias of frail elder subjects. All participants underwent a medical examination, physical and cognitive test, and a questionnaire. The physician had access to medical records. To not speak Swedish was the only exclusion criteria.

### Urine incontinence

Self-reported questionnaires were used to obtain data of UI. The participants were to answer if they had problem of urine leakage and if so, how frequently, the four alternatives being: daily, 2–3 times a week, once a week and occasionally a month. Any UI included all four categories. Questions included ever use of aid for UI (e.g., absorbent products) or if participants currently had urine catheter. The frequence and severity of UI in the past three months was graded from not at all, a little, quite a lot and a lot. The categories ‘a little ‘ and ‘a lot’ of UI the past three months were used to classify the prevalence of UI.

### Fecal incontinence

As with UI self-reported questionnaires were used to obtain data on FI. The participants were to answer if they had problem of fecal leakage or loose stools. Those who answered positive how frequently, alternatives being sometimes (once a week) or often (more than once a week) and the use of aid for FI (e.g., diapers, stomia). The severity of FI in the past three months with only fecal leakage was graded from not at all, a little, quite a lot and a lot. The categories ‘a little ‘ and ‘a lot’ of FI the past three months were used to classify the prevalence of FI.

### Statistical analyses

Descriptive statistical analysis with chi square statistics and one-way ANOVA were performed using SPSS 25 of subjects who had responded to the questionnaire. A multivariate logistic regression model was performed with UI and FI as dependent variables adjusted for stroke and dementia. Information on stroke was retrieved from medical records and defined from ICD-codes I160-I169 and dementia was defined according to DSM-5 criteria from medical examination, cognitive assessment and medical records.

### Ethics

This study, including both the earlier- and the later-born cohort, was conducted in accordance with the Declaration of Helsinki, and was approved by the Regional Ethics Committee at Lund University in 2002 (registration no. LU 744-00). All participants provided written informed consent to participate in the study, and for the retrieval of information from the National Patient Register and medical records. Participants were informed that they could withdraw from the study at any time.

## Results

Prevalence of any UI among men 60–69, 70–79 and over 80 years old were 5%, 12% and 18% respectively, and corresponding values for UI the last 3 months were 10%, 17% and 28%, respectively, Table 1. In men over 80 years old 5% were using aids for UI.

**Table 1:** Prevalence and severity of urine incontinence (UI) and fecal incontinence (FI) in the cohort examined 2001

Age	60–69 years old		70–79 years old		Over 80 years old	
Sex	Men	Women	Men	Women	Men	Women
Number of individuals	694	689	251	308	350	639
Responders	669	669	239	296	320	560
*Any UI*	5.2%	18.7%	11.7%	21.3%	18.2%	40.6%
Daily	0.6%	7.2%	2.5%	11.8%	6.9%	24.7%
2–3 times a week	1.0%	1.6%	1.7%	3.7%	2.8%	5.5%
Once a week	0.4%	2.2%	1.3%	0.7%	1.6%	2.1%
Couple times a month	3.1%	7.6%	6.3%	5.1%	6.9	8.0%
*Experienced UI past 3 month*	10.1%	21.2%	16.9%	29.6%	27.5%	41.8%
A little	9.2%	18.2%	14.8%	24.5%	20.6%	27.6%
Quite a lot	0.8%	2.1%	1.3%	4.8%	3.8%	8.0%
A lot	0.2%	0.9%	0.8%	0.3%	2.9%	6.2%
Urine catheter	0.1%	0.3%	0.4%	0	0.6%	0.5%
Aid for UI (e.g. diapers)	0.6%	8.2%	2.5%	10.5%	5.3%	24.5%
*Any FI*	6.5%	7.9%	11%	11.8%	12.6%	18.7%
Sometimes (once a week)	5.7%	6.0%	8.5%	10.1%	8.5%	14.5%
Often (more than once a week)	0.8%	1.9%	2.5%	1.7%	4.1%	4.1%
Aid for FI (e.g. diapers)	0.2%	0.8%	2.1%	2.4%	5.4%	10.7%
*Experienced FI past 3 month*	2.4%	3.9%	7.2%	8.5%	8.3%	14.3%
A little	2.0%	3.4%	6.8%	7.2%	5.4%	9.2%
Quite a lot	0.4%	0.4%	0.4%	1.4%	1.9%	2.9%
A lot	0	0.1%	0	0	1.0%	2.2%

Prevalence of any UI among women 60–69, 70–79 and over 80 years old were 19%, 21% and 41% respectively and corresponding values for UI the past 3 months were 21%, 30% and 42%, respectively. The severity of UI defined as quite a lot or lot of experience ranged from 3% in 60–69 years old to 14% in women over 80 years of age. High severity of UI defined as daily UI was reported by 25% of women and 7% of men.

Prevalence of any FI, including loose stool increased from 7% in men aged 60–69 years to 13% in men over 80 years of age and corresponding values for women were 8% and 19%, see Table 1. High severity of FI defined as more than one weekly were reported by 4% of both men and women in the group over 80 years old. Experience of only fecal incontinence the past 3 month among the 81-year-old cohorts increased from 4% to 11% among the later born men and respectively 12% to14% for the women.

Table 2 presents the four birth cohorts of 60- and 81-year-old men from years 2001 to 2019. No birth cohort difference in UI or FI the past 3 months and any FI were noted for 60-year-old men and women. Birth cohort effects were noted for 81-year old men and women with a higher prevalence of UI reported by later born (6, 12 and 18 yrs later) cohorts, see Table 2. Any UI for 81-year old men increased from 11% year 2001 to 25% among the 2019 cohort (*p* = 0.046) and UI the past 3 months among 81-year old women increased from 32% to 42% in the latest born cohort (*p* < 0.05). OR in the 2019 cohort compared to the 2001 cohort for any UI was 2.95 for men and UI the past months for women 1.61, adjusted for stroke and dementia, see Table 3.

**Table 2:** Prevalence and severity of urine incontinence (UI) and fecal incontinence (FI) in men and women among four different birth cohorts, aged 60 and 81 years respectively, at time of investigation 18 years apart

	60 year old men						81 year old men						
Birth Cohort. year of investigation	2001	2007		2013		2019	2001		2007		2013		2019
Number of individuals	364	533		479		243	107		140		189		193
Responders	347	478		443		208	103		125		168		180
*Any UI* (%) 95% CI	3.7% 1.7–5.7	3.6% 1.5–4.8		3.6% 1.7–5.1		3.4% 0.6–5.2	11.2% 3.9–15.4		21.6% 13.7–28.2		14.3% 8.9–19.6		25.0% 18.6–31.4
Daily	0.9%	0.8%		0.7%		1.4%	1.9%		9.6%		3.0%		7.8%
2–3 times a week	0.9%	0.8%		0.2%		0.5%	3.7%		7.2%		1.8%		6.1%
Once a week	0.3%	0.2%		0.5%		1.0%	0		0.8%		2.4%		3.3%
Couple times a month	1.4%	1.7%		2.3%		0.5%	5.6%		4.0%		7.1%		7.8%
*Experienced UI past 3month* (%) 95% CI	8.9% 5.9–11.9	8.5% 6.0–11.3.0.3		8.4% 5.9–10.9		10.7% 6.7–14.7	19.4% 11.7–27.2		32.2%*** 23.8–40.7		25.3% 18.8–31.8		28.2%* 21.4–34.9
A little	8.0%	8.5%		8.0%		7.7%	16.5%		27.3%		19.5%		25.9%
Quite a lot	0.6%	0		0.2%		2.6%	2.9%		3.3%		4.6%		1.7%
A lot	0.3%	0		0.2%		0.4%	0		1.7%		1.1%		0.6%
Urine katheter	0.3%	0.2%		0		0	2.9%		0.8%		0.6%		0
Aid for UI (e.g. diapers)	0.6%	0.8%		0.2%		1.0%	0.9%		7.2%		3.0%		8.3%
*Any FI* (%) 95% CI	7.6% 4.7–10.3	6.9% 4.6–9.3		4.8% 2.8–6.8		9.2% 5.2–13.1	8.7% 3.2–14.2		16.8% 9.6–22.7		11.3% 6.5–13.3		11.4% 6.6–16.1
Sometimes (once a week)	7.0%	6.0%		3.7%		6.8%	5.8%		11.2%		9.5%		10.2%
Often (more than once a week)	0.6%	0.8%		1.1%		2.4%	2.9%		5.6%		1.8%		1.1%
Aid for FI (diapers etc.)	0	0.6%		0.2%		0.5%	1.0%		5.6%		4.2%		2.8%
*Experienced FI past 3 month* (%) 95% CI	2.3% 0.7–3.9	2.5% 1.2–4.3		2.6% 1.1–4.0.1.0		4.7% 2.0–7.4.0.4	3.9% 0.1–7.8		14.9% 8.4–21.3		9.8% 5.3–14.2		10.9% 6.2–15.6
A little	1.7%	2.1%		2.2%		3.8%	1.9%		11.6%		5.7%		10.3%
Quite a lot	0.6%	0.2%		0		0.9%	1.9%		0.8%		2.3%		0.6%
A lot	0	0.2%		0.4%		0	0		2.5%		1.7%		0

Age and gender	60 year old women							81 year old women					
Birth Cohort. year of investigation	2001		2007		2013	2019		2001	2007	2013		2019	
Number of individuals	356		663		452	215		155	187	230		259	
Responders	336		611		413	195		155	164	191		228	
Any UI (%) 95% CI	17.2% 13.2–21.2		11.5% 8.8–14.2		17.1% 13.5–20.9	11.3% 6.8–15.8		33.5% 25.4–40.4	39.0% 31.3–46.5	39.2% 31.8–45.7		37.7% 31.0–43.6.0.6	
Daily	7.1%		4.9%		5.6%	4.6%		20.0%	21.3%	22.0%		21.1%	
2–3 times a week	1.5%		1.8%		1.7%	1.5%		6.5%	6.7%	3.7%		7.5%	
Once a week	2.1%		0.3%		1.9%	2.0%		1.9%	3.0%	2.6%		2.6%	
Couple times a month	6.5%		4.4%		8.0%	3.1%		5.2%	7.9%	11.0%		6.6%	
Experienced UI past 3mth (%) 95% CI	20.3% 15.8–24.4		15.4% 13.0–19.3.0.3		20.6% 16.8–24.4	19.3% 13.9–24.8		32.3% 24.4–39.3	40.1%*** 31.8–47.6	39.0% 32.4–45.7		42.1%* 35.8–47.6	
A little	17.0%		13.4%		16.5%	15.0%		24.5%	27.6%	28.6%		29.8%	
Quite a lot	2.4%		1.2%		3.2%	3.4%		5.8%	8.6%	7.6%		9.8%	
A lot	0.9%		0.8%		0.9%	1.0%		1.3%	3.9%	2.9%		2.6%	
Urine katheter	0.3%		0		0	0		0.6%	1.2%	0.5%		0.4%	
Aid for UI (e.g. diapers)	7.1%		4.1%		6.3%	4.1%		16.8%	21.3%	20.9%		18.4%	
Any FI (%) 95% CI	8.1% 5.1–10.9		7.9% 5.7–10.3		11.9% 8.8–15.0	10.3% 6.0–14.6.0.6		16.8% 10.9–22.9	12.3% 6.9–16.9	12.1% 7.4–16.8		19.7% 14.5–25.0	
Sometimes (once a week)	6.0%		6.6%		9.5%	8.2%		14.2%	11.1%	8.4%		15.2%	
Often (more than once a week)	2.1%		1.3%		2.4%	2.1%		2.6%	1.2%	3.7%		4.5%	
Aid for FI (e.g. diapers)	0.3%		0.5%		1.0%	1.0%		5.2%	3.1%	5.2%		7.9%	
Experienced FI past 3 mth (%) 95% CI	3.0% 11.2–4.8		4.2% 2.4–5.8		5.2% 3.1–7.3	6.3% 3.0–9.6.0.6		11.6% 6.6–16.8	8.6% 4.1–13.1	8.6% 4.8–12.4		14% 9.6–18.5	
A little	2.1%		3.2%		3.8%	5.3%		9.0%	7.9%	5.2%		10.6%	
Quite a lot	0.9%		0.3%		0.9%	1.0%		1.3%	0.7%	1.4%		2.6%	
A lot	0		0.7%		0.5%	0		1.3%	0	1.9%		0.9%	

* *p* < 0.05 cohort 2001 vs. 2019; *** *p* < 0.001 cohort 2001 vs. 2007

**Table 3:** A multivariate logistic regression model with four birth cohorts 60 and 81 year old at different examination periods as independent variables and urine incontinence (UI) and fecal incontinence (FI) as dependent variables, adjusted for stroke and dementia

	60 year old men 81 year old men				60 year old women 81 year old women			
Independent variables	OR	*p*-value	OR	*p*-value	OR	*p*-value	OR	*p*-value
Number of individuals	1585		620		1603		825	
*Any UI*								
Cohort year 2001	1		1		1		1	
Cohort year 2007	0.86	0.706	2.25	0.044	0.58	0.007	1.28	0.300
Cohort year 2013	0.96	0.908	1.48	0.329	1.06	0.759	1.28	0.284
Cohort year 2019	0.78	0.622	2.95	0.004	0.64	0.093	1.18	0.467
*Experienced UI past 3months*								
Cohort year 2001	1		1		1		1	
Cohort year 2007	1.02	0.939	1.84	0.057	0.76	0.129	1.42	0.153
Cohort year 2013	0.99	0.977	1.39	0.289	1.10	0.610	1.59	0.045
Cohort year 2019	1.28	0.411	1.58	0.135	1.05	0.839	1.61	0.034
*Any FI*								
Cohort year 2001	1		1		1		1	
Cohort year 2007	0.96	0.882	1.91	0.131	0.98	0.939	0.61	0.141
Cohort year 2013	0.61	0.115	1.19	0.695	1.54	0.093	0.66	0.186
Cohort year 2019	1.31	0.396	1.29	0.550	1.38	0.306	1.19	0.519
*Experienced FI past 3months*								
Cohort year 2001	1		1		1		1	
Cohort year 2007	1.19	0.711	3.98	0.016	1.30	0.509	0.70	0.361
Cohort year 2013	0.92	0.865	2.82	0.070	1.72	0.176	0.72	0.364
Cohort year 2019	2.35	0.720	2.95	0.056	2.39	0.043	1.26	0.466

FI the past 3 months among 81-year old men increased from 4% to 11% in the latet born cohort (OR 2.95, *p* = 0.056) and corresponding values for 60-year old women increased from 3% to 6%, OR 2.39, *p* = 0.04, adjusted for stroke and dementia. Only one out of three men and one out of two women with UI used aids.

## Discussion

This study has assessed prevalence and severity of urine and fecal incontinence in elderly general population. The occurrence of both urine and fecal incontinence increases with age and is highly prevalent among elderly men and women. An interesting but somewhat unexpected birth cohort effect was noted, in that there was an increased prevalence of UI in the 81-year-old men and women who were born at a later time (year 1938/40 versus 1919/21). A similar trend was seen for FI for 81-year-old men, being more common in those born more recently.

A recent meta-analysis on prevalence of UI in women including 518 465 individuals from twenty-nine studies reported a high variation across studies, as low as 9.5% in women 60 years and older in Mexico and as high as 80% in Egypt [7]. The three largest studies included were studies from Canada and US where surveys were used to assess prevalence of UI which ranged from 14% to 38% in women 65 years and older and similar to this study [24–26]. The prevalence of UI for women ranged from 19% to 41% for those 80 years and older. In contrast, a study with data from Germany and Denmark, using same age group intervals as in this study, noted higher UI prevalence 46% to 50% in 60–69 years old women and 58% to 61% in those over 80-year of age [9]. That study used the International Consultation on Incontinence Modular Questionnaire Urinary Incontinence Short Form (ICIQ UI-SF) to assess the prevalence of UI and similar to the questions used in this study [27]. Some of the variation in prevalence across studies might be attributed to different definitions of UI, and difference in prevalence of risk factors, but also knowledge of treatment options, access to treatment and patient’s perceived stigmatization can contribute to variation in reported prevalence.

In a systematic review of UI in men, forty-one studies were pooled, and the prevalence was 21% for men older than 65 years. Majority of those studies came from America and Europe and included predominantly white population [8]. However, data are scarce on the incidence of UI in community dwelling men and data for aged groups over 80 year is often lacking. The same factors as for women might explain the large variation across studies.

Only few studies have addressed the prevalence of FI in the elderly and data for women and men is often not separated. In recent publications the prevalence of FI varies from 4% to 18% in community dwelling individuals [1, 10]. A large Swedish postal survey of self-reported FI with the same questions as in this study reported similar findings with 9% to 11% FI among 75–79-year-old men and women compared to this study [1]. Two other large US studies have noted similar FI prevalence in the range of 16 to 18% for individuals 65 to 75 years and older [10, 28]. The SABE study from 2016 presenting prevalence of FI in the population of Brazil is comparable to our study in many ways [4]. Both studies showed an increased prevalence of UI and FI with age and more common in women than in men. This trend that UI is more common in women has been observed in number of other studies, while data on FI is more conflicting [4, 8, 9, 12, 28–31]. One of the questions about FI in this study included both fecal leakage and loose stool that might explain the higher prevalence compared to data on FI the past 3 months that only included a question about fecal incontinence.

Life span and health increases among the oldest old and improvement in survival for 95-year olds born in 1915 compared with 1905 was seen across the whole spectrum of health and functioning in two Danish cohorts [15]. Life course epidemiology was coined in the 1990s to define a field of study interested in early-life and later-life determinants of chronic diseases [32]. A recent publication on effect of birth cohorts including all-cause mortality data from 14 European countries in the period 1971 to 2015 reported a decreasing mortality in more recent birth cohorts, strongest for cardiovascular mortality [33]. Educational inequalities in mortality increased among low educated for more recent birth cohorts, especially for women and Eastern Europe. The concept of birth cohort as a surrogate for general health therefore seems to apply especially for the so called Baby Boom birth cohorts in high income countries. Recent studies on health trends in elder subjects from the general population have reported both improved physical and cognitive function in later born cohorts born during 1940thies and 1950thies compared to subjects born 10 to 20 years earlier [13, 14, 16–21]. A decrease in CVD, stroke and dementia have been noted the recent decades [34, 35]. The US Atherosclerosis Risk in Communities cohort study reported a decrease of incidence rate of stroke by 32% per decade from 1987 to 2017 for both men and women 65 years and older [34]. A parallell decrease of dementia prevalence and incidence has been reported from the Health and Retirement Study (2000–2016) supporting the use of birth cohort to evalute various health determinants [35]. Both these conditions are risk factors for UI and FI.

We have previously presented results from the same birth cohorts from the GÅS analyzed in this study, showing better physical functioning, defined as better walking speed and chair stand, in later born 60 and 81-year old birth cohorts [18, 19]. Also cognitive functioning was better in the later born 81 year old cohort with an 0.56 OR for having a mild cognitive impairment compared to the cohort born 17 years earlier, adjusted for education, life style factors, comorbidity and depressive mood [21]. One might expect that also UI in community dwelling later born elder cohorts should show a reduced prevalence. However, when comparing the four different birth cohorts in this study the later born cohorts of 81-year-old men and women instead had higher prevalence of UI, defined as any UI or experienced UI the past 3 months. Several explanations can be considered. A previous study that characterized the stigma of urinary symptoms reported that people who felt stigmatized for UI has implications for help-seeking and likelihood for treatment [36]. Whether the noted birth cohort difference among elderly men is explained by changes of risk factors or reflect changes of perceived stigmatization where men investigated 2019 more openly address UI and FI and seek help thereby introducing a recall bias remain to be investigated in further studies. We cannot rule out potential link to increased obesity and changes in lifestyle that could influence the higher prevalence among later born cohorts. Furtherrmore, a number of medications like diuretics, antidepressants and high blood pressure medication, can cause urinary incontinence. On the other hand, as mentioned earlier, a decrease in dementia and stroke later decades act in the opposite direction. The participation rates were similar between the four cohorts, and we have no reason to believe that the findings were attributed to selection bias between cohorts. However. the observed prevalence of UI and FI is probably an underestimation of true prevalence since frail elder are more prone to reject participation. Home visits were provided to avoid this kind of selection of the frailest elder. The study design included randomization of both urban and rural areas from the general population study thereby improving generalization of findings.

It was not the aim of this study to investigate risk factors for and type or cause of UI and FI. It can only be speculated to what degree individuals suffering from incontinence could benefit from available treatment options (medications, operations). However, even though almost every second women and every fourth male 80 years and older in this study reported UI the past 3 months only 25% of women and 5% of men use aids. The discrepancy between the high prevalence of UI and FI and use of aids, even with available treatment options, signifies the problem that either a large proportion of the patients choose not to discuss their symptom because of stigma or the inadequacy of the health care system to detect and provide treatment for this clinical condition. The use of aids among individuals with FI shows the same pattern. Some explanation could be that less severe symptoms could be handled by existing treatment strategies for UI like lifestyle modification, pelvic floor exercises and medications without using diapers as aids. More advanced treatment for UI includes botulinum toxin, neuromodulation and surgery [6, 29]. On common risk factor in women is pregnancy and of women reporting fecal incontinence 3 months postpartum, 43% had symptoms 12 years after delivery [37]. With treatment options available studies have shown that only 25% of women with UI seek or receive treatment [6]. Treatment available for FI includes dietary interventions, lifestyle modifications and pharmacologic therapy and optimized stool consistency that are effective in most women with mild symptoms [31, 38].

## Conclusion

Urine and fecal incontinence are highly prevalent in the Swedish general elder population and the prevalence of both UI and FI is higher in women and increases with age. A birth cohort effect is noted with an unexpected increasing prevalence of UI and FI among 81-year old subjects in the later born cohorts, despite a better general health in these birth cohorts, as reported earlier. Further studies are called for to investigate to which extent the findings are explained by perceived stigmatization or inability from the health care system to detect these clinical conditions. Nevertheless, the findings highlight a need for improved public education to increase help seeking behaviour.

## Supplementary Information

Supplementary Material 1

## Data Availability

Data availability statement: Data that support the findings of this study is presented in the paper.
